# Partial Oxidation to Extend the Lifetime of Nanoporous Carbon in an Ultracapacitor with Li_2_SO_4_ Electrolyte

**DOI:** 10.3390/molecules28072944

**Published:** 2023-03-25

**Authors:** Maike Käärik, Mati Arulepp, Jaan Leis

**Affiliations:** 1 Institute of Chemistry, University of Tartu, Ravila 14a, 50411 Tartu, Estonia; 2 Skeleton Technologies, Sepise 7, 11415 Tallinn, Estonia

**Keywords:** nanoporous carbon, carbide-derived carbon, electrical double-layer capacitor, pH neutral electrolyte, aqueous electrolyte

## Abstract

A TiC-derived carbon (CDC) and its partially oxidized derivative (ox-red-CDC), oxidized by a modified Hummers method, were studied as promising electrode materials for electrochemical energy storage. To evaluate the electrochemical properties of the carbon materials, cyclic voltammetry, galvanostatic cycling, and electrochemical impedance spectroscopy measurements were performed in 1 M Li_2_SO_4_ using 2- and 3-electrode cells. A partially oxidized surface was shown to improve the capacitance and electrochemical stability of a nanoporous CDC at positive potential values. The respective anodic capacitance of 80 F cm^−3^ reveals a 15% improvement over the non-oxidized CDC. At negative potential values, the capacitance of two carbon materials is almost equal, 97 vs. 93 F cm^−3^, for the non-oxidized and partially oxidized CDC materials, respectively. An asymmetric 2-electrode ultracapacitor containing ox-red-CDC as the anode and pristine CDC as the cathode demonstrated an excellent cycle life. The temporary repolarization of the 2-electrode cell after thousands of charge–discharge cycles increased the capacitance and improved the cycling characteristics, likely due to regeneration and cleaning of the electrode surface.

## 1. Introduction

Energy storage materials based on nanoporous carbon, despite being extensively studied in recent years, are still an area with a lot of unexplored potential. Among numerous materials, the carbide-derived carbon (CDC) [1] with a well-tuned pore size distribution (PSD) receives much attention as an electrode material [2,3,4,5] for the ultracapacitors [6].

Ultracapacitors (this article mainly discusses the electrochemical double-layer capacitor (EDLC)) are long-life, carbon-based energy storage devices that offer higher power density, faster charge/discharge, and a longer cycle life than batteries. EDLCs store energy through the electrostatic accumulation of charges at the electrode–electrolyte interface; therefore, the larger the specific surface area of the carbon electrode, the higher the capacitance [7]. Typically, ultracapacitors use aprotic organic electrolytes, but due to the global pressure to adopt more environmentally friendly and cheaper energy storage materials, much more emphasis must be placed on the research of aqueous electrolytes, especially electrolytes with a neutral pH level [8].

The feature that makes the CDC interesting for ultracapacitors is the possibility to fine-tune its structure and porosity. More precisely, the properties of CDC depend on the structure and composition of the precursor carbide (e.g., the CDC particles retain the shape and size of the precursor carbide) as well as on the synthesis conditions (e.g., the higher the synthesis temperature, the greater the structural order of CDC) [9]. In addition, CDC’s textural properties and surface functions can be fine-tuned chemically [10,11] or by applying common physical activation methods [12,13,14]. 

The correct pore size distribution of the carbon is a key factor in the high energy density of the supercapacitor: the better the electrolyte ions and pore size match, the higher the achievable capacitance [9,15]. When an aqueous solution is used as the electrolyte, both the strong solvation of ions [16] and possible redox processes [17,18] occur, complicating the interpretation of the relationship between the capacitance and textural properties of carbon. The most widely used pH-neutral aqueous electrolytes are solutions of lithium [19,20,21,22,23,24,25], sodium [26,27,28], and potassium salts [29], as they meet the main requirements for an electrolyte: high ionic conductivity, sufficient chemical, and electrochemical stability, a wide operating temperature range, environmental friendliness, and low costs [13,30]. Here, it is relevant to note that aqueous electrolytes with alkaline salts have a lower operating voltage than non-aqueous electrolytes in Li, Na, or K batteries, which prevents the occurrence of alkali metal intercalation and redox processes in the negatively charged carbon electrode [31,32,33,34].

It has been shown that 1 M Na_2_SO_4_ and 1 M Li_2_SO_4_ result in a similar capacitance of ~120 F g^−1^ for the porous cellulosic carbon electrodes in symmetric double-layer capacitors at a scan rate of 10 mV s^−1^ [35]. However, the capacitance also depends on the potential scan rate. It has been shown that at low scan rates, the capacitance values of activated carbon electrodes do not differ much in 0.5 M Li_2_SO_4_, Na_2_SO_4_, and K_2_SO_4_, but the difference is evident at a scan rate of 100 mV s^−1^ [29]. An additional observation is that when the electrodes have high microporosity, the better mobility of Li^+^ guarantees a significantly higher capacitance for the Li_2_SO_4_ electrolyte compared to potassium and sodium sulfate, even at low scan rates [20]. Another advantage of lithium salts is their better solubility in water compared to potassium or sodium salts, making them attractive in aqueous energy storage research [19,20,22,23,24]. Neutral electrolytes can work at a wider potential window and show very stable behavior in long-term tests, while providing higher power density than, for instance, alkali electrolytes (21 kW kg^−1^ vs. 15 kW kg^−1^) [35].

Previous studies have shown that certain CDCs can be prospective electrode materials for pH-neutral aqueous electrolytes [26,27,28]. The TiC-derived carbon has produced high capacitance values in 1M Na_2_SO_4_ (126 and 134 F g^−1^ for positively and negatively charged electrodes at 2 mA cm^−2^, respectively) [28]. It was demonstrated that CDC electrodes derived from Mo_2_C have a high charge-discharge efficiency (98%) in 1 M Na_2_SO_4_ at U ≤ 1.0 V [26].

The biggest challenge of ultracapacitors with aqueous electrolytes is the charge–discharge cycling stability and sustainability of carbon electrodes. One way to increase the durability of carbon electrodes is to chemically passivate the carbon surface, e.g., using moderately oxidized carbon material in the electrodes. The preoxidized carbon surface improves the wettability of initially hydrophobic carbon electrodes. In addition, it reduces the probability of the electrochemical oxidation of carbon in an aqueous electrolyte, thus allowing an increase in the working voltage and the energy density of the respective ultracapacitor. It has been shown that the deactivation of the carbon surface by chemical oxidation with hydrogen peroxide prevents the positive carbon electrode from electro oxidation, achieving an excellent cycle life of over 10,000 cycles in a 1.9 V potential window using 2 M Li_2_SO_4_ as the electrolyte [23]. The oxidation by the modified Hummers method [36] was shown to improve the wettability of microporous carbon, which resulted in a 16-fold increase in the capacitance of the oxidized CDC up to 228 F g^−1^ in 6 M KOH with an excellent cycling stability [37]. Our recent work showed that partially oxidized CDC is also a beneficial anode material in pH-neutral electrolytes, as confirmed by a series of experiments with ultracapacitors using 1 M Na_2_SO_4_ electrolyte [28].

The aim of this work is to investigate the effectiveness of oxidative protection for microporous carbon in a Li_2_SO_4_ electrolyte. As discussed above, electrolytes with lithium salts tend to provide a higher double-layer capacitance than sodium salts due to the different ion sizes of Li^+^ and Na^+^, suggesting that TiC-derived ultra-microporous carbon electrodes would be particularly suitable for high energy density with small lithium cations. Moreover, this study is of particular interest because, to our knowledge, no results have been published on the electrochemistry of CDC-type carbon materials (except low-density aerogel [38]) in a Li_2_SO_4_ electrolyte.

## 2. Results and Discussion 

**Figure 1:**
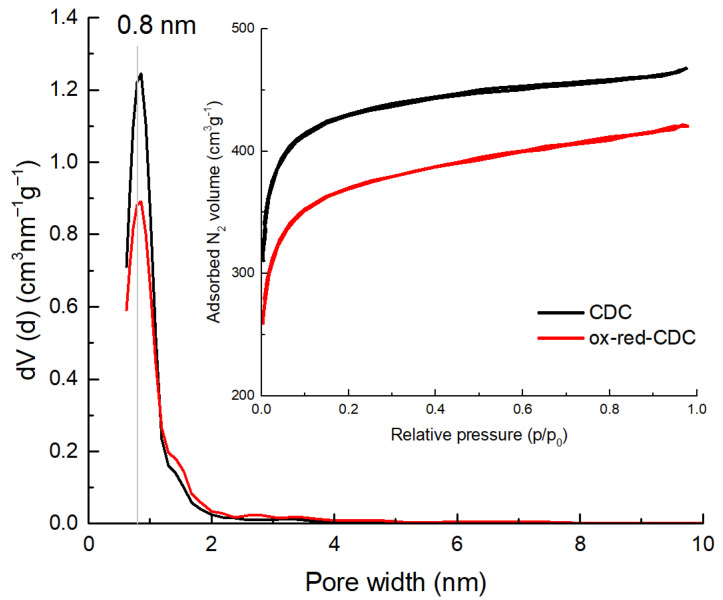
Pore size distribution of CDC and ox-red-CDC calculated from N_2_ isotherms (inset).

The electrochemical properties and energy storage capability of two nanoporous carbon materials were investigated and compared to each other using 1 M Li_2_SO_4_ aqueous solution as an electrolyte. One of the carbon materials was obtained directly from titanium carbide using a high-temperature chlorine treatment (denoted here as CDC) and the other was its surface-modified derivative, further oxidized using a modified Hummers method (denoted as ox-red-CDC). The details of the synthesis and chemical treatment conditions were published previously [28]. The main difference between the two materials, as thoroughly discussed in our previous paper, is that ox-red-CDC has a higher surface oxygen content (5.2 ± 0.6 wt%) compared to the original CDC (3.4 ± 0.9 wt%), with a significantly higher proportion of C=O groups in ox-red-CDC [28]. Slight differences can also be found in pore size distribution (Figure 1) and the values of textural properties (Table 1). As can be seen from the isotherm graph (Figure 1, inset), both materials are predominantly microporous materials with Type I isotherm by IUPAC [39]. Both the materials have a narrow pore size distribution with a maximum of 0.8 nm, whereas pristine CDC had a slightly higher specific surface area, i.e., 1500 and 1300 m^2^ g^−1^, respectively.

**Figure 2:**
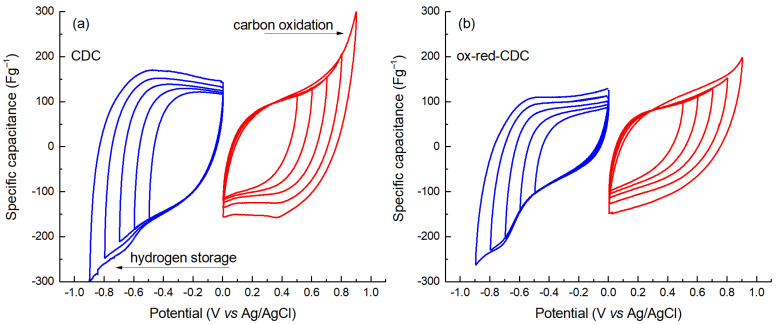
CV curves (*v* = 2 mV s^−1^) for positively and negatively charged electrodes of CDC (**a**) and ox-red-CDC (**b**).

The capacitance of CDC and ox-red CDC at opposite polarization potentials was studied by constant current (CC, 2 mA cm^−2^; 0 V to +/− 0.75 V) and cyclic voltammetry (CV, 0 V to +/−0.9 V by 0.1 V step) methods using a 3-electrode cell with a Ag|AgCl reference electrode. The CV curves for positively and negatively polarized CDC material are presented in Figure 2, reflecting the typical behavior of EDLC materials with good charge propagation. As usual with microporous electrodes, the capacitance of both materials shows a dependence on the potential window—the wider the window, the higher the capacitance. Such a phenomenon is explained by the fact that in a stronger electric field, the dimensions of the ions located in the electric double layer decrease and, therefore, their number per unit area increases—more ions can fit into small-sized micropores. Comparing the curves of the CDC and ox-red-CDC materials at positive potential values (Figure 2a,b, respectively) reveals that the voltage stability of ox-red-CDC is better. At the same time its capacitance does not increase significantly with increasing potential. Unlike CDC, the ox-red-CDC electrode does not show carbon oxidation-reduction peaks up to the cycle interval of 0 to 0.8 V (vs. Ag|AgCl). On the other hand, the shape of the CV curves at the negative potential values is quite similar for both materials, indicating a sharp increase in the charging currents below –0.6 V (vs. Ag|AgCl). Thus, it can be said that the protection of microporous carbon by oxidation, as in ox-red-CDC, is particularly suitable for the positive electrode material of EDL capacitors in the case of a Li_2_SO_4_ electrolyte.

Cells were preconditioned with 1000 charge–discharge cycles at 20 mV s^−1^ at a potential range of 0 V to +0.5 V or 0 V to −0.5 V (vs. Ag|AgCl), depending on whether a positively or negatively polarized electrode was tested. The capacitance values are presented in Table 2. The highest gravimetric cathodic capacitance ($C_{cc}^{-}$ 150 F g^−1^) was achieved in the case of CDC, which has a higher fraction of micropores below 1 nm compared to ox-red-CDC (cf. Figure 1). Interestingly, there is no difference in the anodic capacitance between the materials, which indicates that for the electrosorption the SO_4_^−^ anion of the electrolyte requires slightly larger pores than Li^+^. The pore size distribution of CDC and ox-red-CDC (Figure 1) confirms that CDC has a significantly larger pore volume of around 0.8 nm, while a >0.9 nm pore volume is almost the same.

If we compare the capacitances measured in Li_2_SO_4_ and Na_2_SO_4_ [28] electrolytes, it appears that the cathodic capacitance (i.e., at negative potentials) of both carbon materials, TiC-derived CDC and its oxidized derivative, ox-red-CDC, is significantly higher in the Li_2_SO_4_ electrolyte, 150 vs. 134 F g^−1^ and 132 vs. 120 F g^−1^, respectively. The higher capacitances with Li_2_SO_4_ electrolyte are consistent with the size of the bare cations (Li^+^ < Na^+^) although the diameter of the hydrated Li^+^ ion is larger than the diameter of the hydrated Na^+^ ion (0.76 nm vs. 0.72 nm) [29,40].

The density of ox-red-CDC electrode is higher than that of pristine CDC: 0.80 vs. 0.72 g cm^−1^, respectively, which is most probably due to the improved adhesion of ox-red-CDC particles with a modified surface structure. The data represented in Table 2 reveal that the volumetric capacitance of CDC and ox-red-CDC, which is directly impacted by the electrode density, is equal in the case of negative polarization. In contrast, at a positive polarization, the capacitance of the oxidized material, ox-red-CDC, exceeds the capacitance of the pristine CDC by 18%. Given the growing interest and practical need for electrode materials with volumetric capacitance [41], this is an important finding that contributes to a better understanding of the factors affecting volumetric capacitance.

Previously, we have reported that the partially oxidized CDC as an anode material improves the electrochemical stability of an ultracapacitor with the aqueous Na_2_SO_4_ electrolyte. The Li_2_SO_4_ electrolyte is expected to further improve the capacitive characteristics of the unsymmetric ultracapacitor with the pristine CDC as a cathode and the ox-red-CDC as an anode.

**Figure 3:**
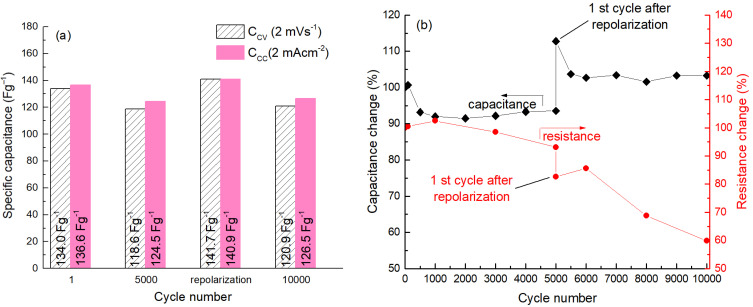
The specific capacitance by CC and CV (**a**), and retention in capacitance and resistance (**b**) of 2-electrode cell at 1.5 V.

Charging–discharging of the unsymmetrical ox-red-CDC/CDC cell up to 10,000 cycles was performed using the CV method at a scan rate of 20 mV s^−1^ in the voltage range of 0 to 1.5 V (see Figure 3). The polarities of the current terminals of the cell were reversed after 5000 cycles, followed by 10 CV cycles between 0 and 1.5 V. The original polarities were then restored and the cycling continued. Such a procedure with shortly reversed electrode potentials (here called “repolarization”) caused a significant change in cell characteristics (i.e., *C* and *R*). For example, after repolarization at 1.5 V, the capacitance exceeded an even higher value than initially observed. The increase in capacitance after repolarization is explainable due to the reorganization of ion pairs on the carbon surface. Immediately after repolarization, the amount of free ions at the electrode/electrolyte interface is larger until a new equilibrium is reached. In addition, during repolarization, the so-called cleaning of the carbon surface occurs, i.e., electroactive compounds that have blocked a part of the carbon surface due to the electric field (or are weakly adsorbed there) desorb from the surface into the solution, and restructuring takes place in the carbon micropores. A similar phenomenon has been described by Piwek et al. [25].

The capacitance increase due to brief repolarization was observed at both 20 and 2 mV s^−1^ sweep rates. As we can see from Figure 3b, after repolarization, the capacitance quickly decreases by about 10% (from 83 to 76 F g^−1^) within the next 500 cycles, but then it remains very stable, up to 10,000 (overall) cycles tested. After that, the cycling interval was increased by 0.1 V to the operating voltage of 1.6 V for the next 5000 cycles. However, unfortunately, the rapid loss of the capacitance observed already after 2000 cycles reveals that the voltage window of 0–1.6 V is too high for this electrochemical system. However, the effect of repolarization applied after the 5000th cycle was also manifested when cycling at an operating voltage of 1.6 V. This demonstrates the potential importance of regular repolarization in extending the cycle life of an ultracapacitor.

**Figure 4:**
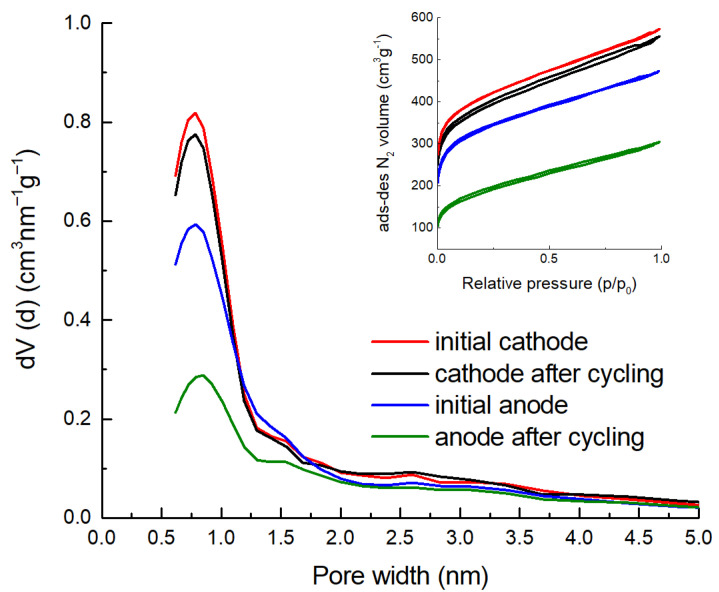
Pore size distribution of cathode and anode before and after cycling calculated per weight of CDC from N_2_ isotherms (inset).

The reduction in cell capacitance during cycling is mainly caused by the destruction of the positive electrode. Since the specific capacitance of the cathode (negative electrode) is almost 20% greater than that of the anode (see Table 2), in the case of an unbalanced pair of electrodes, the anode operates in a wider potential range than the cathode. In particular, when the operating voltage is increased to 1.6 V, the anode potential definitely rises above 0.8 V, which leads to an intense carbon oxidation, which causes the micropores to become blocked, the specific surface area to decrease, and, consequently, the capacitance to decrease. The decrease in specific surface area and the volume of micropores in the positive electrode is confirmed by post-mortem porosity analysis (Figure 4). While in the case of the cathode, only a minimal reduction in the specific surface area (*S*_dft_) and micropore volume (*V*_µ_) per weight of carbon was detected at the end of the cycling test, from 1447 to 1381 m^2^ g^−1^ and from 0.55 to 0.53 cm^3^ g^−1^, respectively, for the anode these values were almost halved, i.e., from 1157 to 611 m^2^ g^−1^ and from 0.46 to 0.24 cm^3^ g^−1^.

**Figure 5:**
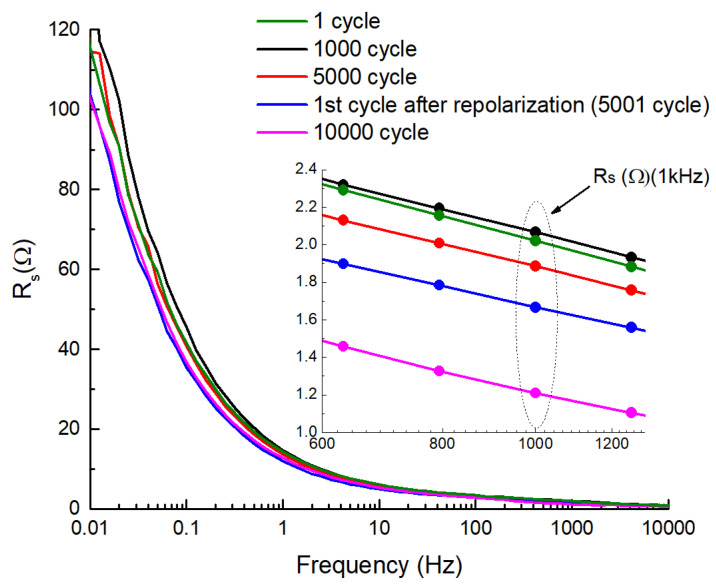
Series resistance *R_s_* vs. frequency of the 2-electrode cell at 1.5V.

The electrochemical impedance spectroscopy measurements were carried out in the frequency range of 1 MHz to 5 mHz (see Figure 5). The *R* values measured at 1 kHz were used to characterize the changes in the internal resistance of the cell during the cycle-life test. As we can see in Figure 3b, in contrast to normal aging, the internal resistance of the cell does not increase during cycling, but instead decreases relative to the original resistance. Repolarization apparently has a “refreshing” effect on the carbon/electrolyte interface and immediately lowers the internal resistance of the cell (see Figure 3b) by approximately 11%. The next few cycles then increase the internal resistance slightly, which continues to slowly decrease thereafter.

**Figure 6:**
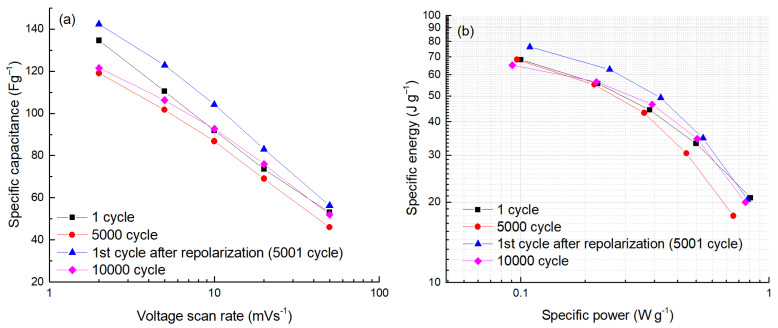
The dependence of specific capacitance on scan rate (**a**) and specific energy vs. specific power (**b**) measured during the cycle-life test at 1.5 V.

**Figure 7:**
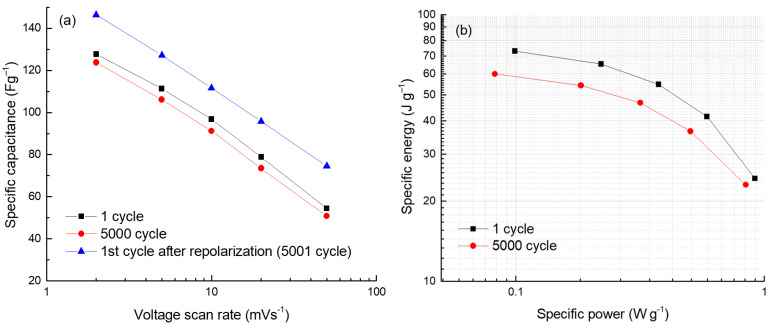
The dependence of specific capacitance on the voltage scan rate (**a**) and a plot of specific energy vs. specific power (**b**) measured during the life-cycle test at an operating voltage of 1.6 V.

During lifetime testing, the capacitance change of the cell was evaluated at several CV scan rates between 2 and 50 mV s^−1^. In Figure 6a, the results are presented on the log scale of scan rates, which gives an almost linear capacitance dependence as characteristic of the EDLC. Some deviation from linearity at low scan rates, observed after 5000 cycles (see Figure 6a), could be explained as an effect of a partial clogging (sieving) of a nanoporous network of the carbon. This “clogging” effect is reduced after cleaning the surface by repolarization (compare the purple and blue curves in Figure 6a). A similar trend was also seen at a higher voltage of 1.6 V (see Figure 7a).

The dependence of the energy vs. power, or the so-called Ragone plot, compiled from the data of CV measurements (see Figure 6b), expresses the energy storage capability of the unsymmetric ultracapacitor with respect to the cycling lifetime, including the repolarization effect. At low power densities (<0.2 W g^−1^), the curves of the Ragone plot coincide for the ultracapacitor at the beginning of cycling and after 5000 cycles (Figure 6b), indicating that even after 5000 cycles of full discharge at 1.5 V, the energy capacity of the carbon electrodes is very stable. However, a slight decrease in power density is seen after 5000 cycles (0.84 vs. 0.68 W g^−1^ at 20 J g^−1^), due to the reduction in material capacitance at shorter discharge times. The repolarization increases the capacitance and energy density of the ultracapacitor. During the next 5000 cycles after repolarization, there is a significant energy reduction of 76 vs. 65 J g^−1^ on the power scale of ~0.08 W g^−1^. At higher power densities (>0.6 W g^−1^), the Ragone curves intersect due to the significant reduction in internal resistance after repolarization. The decrease in the resistance of the ultracapacitor leads to an increase in energy at high power densities.

The Ragone curves were also measured during cycling in the voltage window of 1.6 V (Figure 7b). A comparison of the energy densities after the first and 5000^th^ cycles reveals the energy drop from 73 to 59 J g^−1^ at the power density of 0.1 W g^−1^. This is a much faster change than observed during cycling at 1.5 V. Since the internal resistance of the cell also increases during cycling at 1.6 V (see Figure 7b), the drop in power density during cycling is significant, confirming that the 1.5 V operating voltage should not be exceeded for this particular electrochemical system.

## 3. Materials and Methods

### 3.1. Synthesis, Modification, and Characterizations of CDC Material

Nanoporous TiC-derived carbon (CDC) was made by a gradual chlorination of carbide (TiC, H.C. Starck, Ø < 4 µm, 75 g) at various temperatures [42], the CDC powder was chemically oxidized by Hummers method [36] and finally the dry carbon powder was reduced with hydrogen.

(1) $$TiC+2Cl_{2}\frac{950-800\;{}^{\circ}C}{-TiCl_{4}}>\text{CDC}\;\;\frac{1.Hummer^{\prime }oxidation}{2.Hydrogen\;reduction}>\text{ox-red-CDC}$$

The detailed synthesis conditions and physicochemical characterizations of CDC and ox-red-CDC powder are described elsewhere [28].

The porosity parameters of the carbon materials were determined by low-temperature N_2_ adsorption using a NOVAtouch LX2 (Quantachrome Instruments, Boyton Beach, FL, USA). The electrodes were made by mixing the carbon powder with 10 wt% PTFE (Aldrich, 60% dispersion in water), which was then pressed into a film with a thickness of 100 ± 10 µm. The density of the electrodes was 0.72 and 0.80 g cm^−3^ for CDC and ox-red-CDC, respectively. The carbon film of the counter electrode was prepared from high specific surface area activated carbon powder (V2, EnerG2 Technologies Inc., Seattle, WA, USA), using the same method described above. Before assembling the test cells, the carbon electrodes were kept under vacuum at 110 °C for 48 h. 

### 3.2. Electrochemical Characterization

The electrochemical measurements were performed using cyclic voltammetry (CV), galvanostatic, i.e., constant current (CC) methods and electrochemical impedance spectroscopy (EIS) using the potentiostat-galvanostat 1286 with FRA 1255B (Solartron).

The characterization of carbon materials was carried out in 3-electrode test cells with gold current collectors and a Ag|AgCl (3.5 M KCl) reference electrode (RE). The discs of the working electrode (WE, Ø 7 mm) and counter electrode (CE, Ø 16 mm) were separated by a glass fiber separator (Whatman, thickness 1 mm). A 1 M Li_2_SO_4_ (Alfa Aesar, anhydrous, 99.7%) in Milli-Q water as the electrolyte was used in all experiments. Prior to measurements, the electrodes in the cell were soaked in the electrolyte for approximately 24 h. Firstly, 3-electrode cells were preconditioned with 1000 CV cycles (*v* = 20 mV s^−1^) from 0 to +/−0.5 V vs. Ag|AgCl, according to the selected anode or cathode polarization to achieve the reproducibility of the measured data.

The CC measurements with positively and negatively charged electrodes were carried out with a current of 2 mA cm^−2^ (~0.3 A g^−1^) from 0 to +/−0.75 V vs. Ag|AgCl. To ensure a full charging of cells in all CC experiments, the test cell was kept at a constant potential (+/−0.75 V vs. Ag|AgCl) for 5 min before discharge. The specific capacitances of the carbon materials *C*_CC_ are given for the mass of carbon in the working electrode. The CV curves of CDC and ox-red-CDC at various potential windows up to an interval of −0.9 V to +0.9 V were measured at a scan rate of 2 mV s^−1^. The anodic (0 to +0.9 V vs. Ag|AgCl) and cathodic (0 to −0.9 V vs. Ag|AgCl) performance of the materials was tested separately to avoid the repolarization of the carbon material during the experiment.

**Figure 8:**
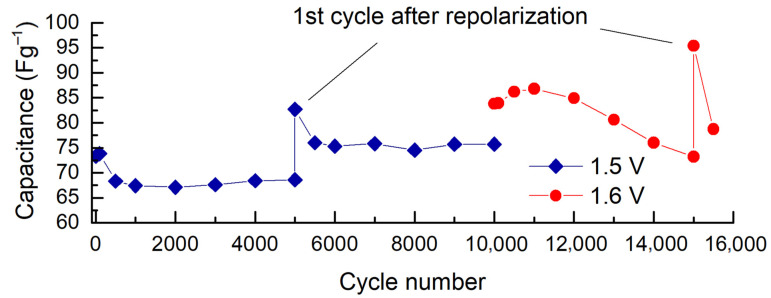
Cycle-life test profile with an unsymmetrical 2-electrode cell at a voltage scan rate of 20 mV s^−1^ including the repolarization and operating voltage change.

The cycling stability of the carbon materials was evaluated in an unsymmetrical cell, which was assembled from two 7 mm diameter electrodes (with ox-red-CDC as the anode and CDC as the cathode) attached to gold current collectors and separated with a Whatman separator (thickness 1 mm). The CV applying a voltage scan rate of 20 mV s^−1^ was used to evaluate the cycling life in the voltage ranges of 0–1.5 V and 0–1.6 V (Figure 8). To assess the effect of the repolarization, the polarity of the cell terminal was reversed after the 5000th cycle, followed by ten CV cycles from 0 to 1.5 V. The original polarity of the cell terminal was then restored, and the cell was charged–discharged for another 5000 cycles (500 cycles in the case of 1.6 V operating voltage) using a voltage sweep rate of 20 mV s^−1^. Prior to the test, the cell was preconditioned with CV cycling (*v* = 50 mV s^−1^), expanding the potential window from 0 to +/−2.0 V vs. Ag|AgCl gradually with an interval of 0.1 V. The specific capacitance of an unsymmetrical cell was expressed per mass of carbon in one electrode as *C* = 4 × (*C*_cell_/*m*_C_)_,_ where *m*_C_ is the total mass of the active carbon in the cell. The CV data were used to construct the experimental Ragone plots (energy density vs. power density). The EIS measurements, to observe the changes in resistance, were performed in the frequency range of 1 MHz to 5 mHz at an AC voltage of 5 mV, using the ZView ver. 3.5i software (Scribner Associates Inc., Southern Pines, NC, USA).

## 4. Conclusions

For the first time, the electrochemical properties and behavior of TiC-derived carbon (CDC) and its partially oxidized derivative (ox-red-CDC) were evaluated and compared in an aqueous Li_2_SO_4_ electrolyte. While the CDC was synthesized from titanium carbide powder using a conventional high-temperature chlorination method, ox-red-CDC was obtained by the oxidation of the same CDC using a modified Hummers method followed by reduction with hydrogen. Both materials were characterized by a high content of micropores with a narrow pore size distribution at 0.8 nm, whereby the pristine CDC had slightly higher specific surface area, i.e., 1500 and 1300 m^2^ g^−1^, respectively. Electrochemical characterization, using cyclic voltammetry, galvanostatic cycling, and electrochemical impedance spectroscopy, was performed in 1 M Li_2_SO_4_. It was shown that the partially oxidized carbon surface has a major impact on the energy storage characteristics of a positively charged electrode, improving its oxidation resistance and increasing the volumetric capacitance. The anodic capacitance of ox-red-CDC is significantly higher than that of a pristine CDC—80 vs. 68 F cm^−3^—probably due to a better adhesion between surface-modified ox-red-CDC particles. At the negative potential values, the capacitance of two carbon materials is almost equal, 97 vs. 93 F cm^−3^. An asymmetric 2-electrode ultracapacitor, containing ox-red-CDC as the anode and pristine CDC as the cathode, showed excellent cycle life, exceeding 10,000 charge–discharge cycles at an operating voltage of 1.5 V. Temporary repolarization of the ultracapacitor after 5000 charge–discharge cycles notably increased the capacitance and improved the cycling characteristics, likely due to the regeneration and cleaning of the electrode surface. In summary, this study demonstrated that partially oxidized CDC is an excellent anode material for ultracapacitors with a pH-neutral Li_2_SO_4_ electrolyte, which shows stable cycling properties and high volumetric capacitance.

## Figures and Tables

**Table 1:** Specific surface area (*S*_BET_ and *S*_dft_), total pore volume (*V*_t_), and volume of micropores (*V*_µ_) calculated from N_2_ adsorption.

Carbon	*S*_BET_	*S*_dft_	*V*_t_	*V*_µ_
Sample	(m^2^ g^−1^)	(m^2^ g^−1^)	(cm^3^ g^−1^)	(cm^3^ g^−1^)
CDC	1499	1511	0.73	0.63
ox-red-CDC	1300	1285	0.65	0.54

**Table 2:** Specific capacitance of CDC and ox-red-CDC by CC at negative and positive potential.

Carbon Sample	$C_{cc}^{-}$ (F cm^−3^)	$C_{cc}^{+}$ (F cm^−3^)	$C_{cc}^{-}$ (F cm^−3^)	$C_{cc}^{+}$ (F cm^−3^)
CDC	150	105	97	68
Ox-red-CDC	132	108	93	80

## Data Availability

All data relevant to this publication are included.
